# Draft Genome Sequence of *Roseomonas mucosa* Strain AU37, Isolated from a Peripheral Intravenous Catheter

**DOI:** 10.1128/genomeA.00128-17

**Published:** 2017-04-13

**Authors:** Md Abu Choudhury, Alexander M. Wailan, Hanna E. Sidjabat, Li Zhang, Nicole Marsh, Claire M. Rickard, Mark R. Davies, David J. McMillan

**Affiliations:** a Alliance for Vascular Access Teaching and Research, Menzies Health Institute Queensland, Griffith University, Brisbane, Australia; b Inflammation and Healing Research Cluster, School of Health and Sports Sciences, University of the Sunshine Coast, Sippy Downs, Brisbane, Australia; c The University of Queensland, UQ Centre for Clinical Research, Royal Brisbane and Women’s Hospital Campus, Brisbane, Australia; d Royal Brisbane and Women’s Hospital, Brisbane, Australia; e Department of Microbiology and Immunology, Peter Doherty Institute for Infection and Immunity, University of Melbourne, Melbourne, Victoria, Australia

## Abstract

*Roseomonas mucosa* is an opportunistic pathogen that causes infections in humans and is often associated with vascular catheter-related bacteremia. Here, we report the draft genome sequence of *Roseomonas mucosa* strain AU37, isolated from a peripheral intravenous catheter tip.

## GENOME ANNOUNCEMENT

*Roseomonas mucosa* is a nonfermenting opportunistic pathogen belonging to the newly established genus of *Roseomonas*. *R. mucosa* is characterized as a fastidious, aerobic, oxidase-positive, and Gram-negative rod which forms mucoidal, almost runny, and pink-pigmented colonies (1). Initially grouped with *Roseomonas gilardii*, *R. mucosa* was designated a new species in 2003 (1). The natural reservoir of *Roseomonas* spp. remains unknown. However, *Roseomonas* spp. have been recovered from environmental sources, such as water and soil (2, 3), as well as multiple sterile and nonsterile clinical sites, including blood, wounds, peritoneal dialysis fluid, genitourinary sites, corneal scrapings, and bone (1, 4 – 7). Although *Roseomonas* spp. generally have low pathogenicity with regard to human infection, some species have been reported to cause clinically significant or even fatal diseases in immunocompromised patients (4). *R. mucosa* has also recently been reported to be associated with vascular catheter-related bacteremia (8, 9).

Genomic analysis of clinical *Roseomonas* isolates is rare. Here, we present the genomic sequence of *R. mucosa* strain AU37, isolated from a peripheral intravenous catheter (PIVC) tip that was positioned in the left arm of 53-year-old male patient admitted at the Royal Brisbane and Women’s Hospital (RBWH). Prior to PIVC insertion, the skin was decontaminated with alcoholic chlorhexidine gluconate. The PIVC was left in place for 7 days and secured with standard polyurethane dressing. The patient was treated with cephalosporin antibiotics during the period of PIVC dwell for a condition unrelated to the PIVC and did not display any systemic infection with *R. mucosa* or other bacteria. After removal, the AU37 PIVC tip was cultured by rolling the tip back and forth on the surface of a blood agar plate, according to the method of Maki et al. (10).

The *R. mucosa* paired-end whole-genome library was prepared via Illumina Nextera XT and sequenced via the Illumina HiSeq 2000, with a read length of 100 bp. *De novo* assembly was performed with the Shovill pipeline version 0.2, which uses SPAdes version 3.9.0 (https://github.com/tseemann/shovill). The resulting 249 contigs (>500 bp), with an *N*_50_ of 48,204 bp, were initially annotated using RAST (http://rast.nmpdr.org) (11). The draft genome of *R. mucosa* (AU37) consisted of 4,741,868 bp (coverage, >~80×), with a G+C content of 70.5%. A total of 4,331 coding sequences (CDSs) were predicted to be present in the genome, 3,353 (79%) of which were assigned putative functions. Eight hundred eighty (21%) CDSs were annotated as hypothetical proteins. The genome contains multiple genes encoding multidrug antibiotic resistance proteins, beta-lactamase and other penicillin-binding proteins, tripartite multidrug resistance, quaternary ammonium compound resistance, organic solvent tolerance proteins, as well as copper and arsenical resistance proteins.

These data confirm that *R. mucosa* AU37 possesses genetic determinants predicted to confer resistance to antibiotics commonly used in hospitals and has increased tolerance to antiseptics used for skin decolonization at catheter insertion sites. These findings have implication for the appropriate clinical management of *Roseomonas* infection. The genomic information of the *R. mucosa* AU37 will provide insights into molecular mechanisms underpinning the virulence of this opportunistic pathogen and the acquisition of antibiotic resistance in this species.

### Accession number(s).

This whole-genome shotgun project has been deposited at DDBJ/ENA/GenBank under the accession no. LLWF00000000. The version described in this paper is version LLWF02000000. The BioProject no. is PRJNA298945, and the BioSample no. is SAMN04169051.
